# Migrant women caregivers’ experiences in end-of-life formal care

**DOI:** 10.1590/1980-220X-REEUSP-2023-0031en

**Published:** 2023-07-28

**Authors:** María José Fuentes Kramar, María Paz García-Caro, Alba Mateo Ternero, Celia Martí-García

**Affiliations:** 1 Universidad de Santiago de Chile, Hospital Barros Luco, Santiago de Chile, Chile.; 2 Universidad de Granada, Facultad de Ciencias de la Salud, Departamento de Enfermería, Granada, España.; 3 IB Salut. Centro de Salud, Son Rutlan, Palma de Mallorca, España.; 4 Universidad de Málaga, Facultad de Ciencias de la Salud, Departamento de Enfermería, Málaga, España.

**Keywords:** Caregiver, Emigrants and Immigrant, Hispanic or Latin, Terminal Car, Qualitative Research, Cuidadores, Emigrantes e Imigrantes, Hispânico ou Latino, Cuidados terminais, Pesquisa qualitativa, Cuidadores, Emigrantes e Inmigrantes, Hispánicos o Latinos, Cuidado Terminal, Investigación Cualitativa

## Abstract

**Objective:**

To describe and understand the experience of Latin American migrant women as caregivers of elderly people in situations of advanced illness and end of life.

**Method:**

Qualitative study using Gadamer’s hermeneutic phenomenology. Data were collected in 2019 through 9 semi-structured interviews with Latin American women caregivers, who had cared for people at the end of life, in the Province of Granada (Spain).

**Results:**

Two themes emerged: “Migrant caregiver at the end of life” and “And now, what should I do?”: the impact of the loss at the economic, emotional and labor level

**Conclusion:**

Care during the end of life of the cared person generates an additional overload to the situation of migrant women. The experience of this stage is related to the bond with the persons cared and their families, which may affect the development of complicated grief and personal problems related to the loss of employment and the absence of economic support.

## RESUMEN

**Objetivo:**

Describir y comprender la experiencia de las mujeres migrantes latinoamericanas como cuidadoras de personas mayores en situación de enfermedad avanzada y final de la vida.

**Método:**

Estudio cualitativo desde la fenomenología hermenéutica de Gadamer. Los datos fueron recogidos en 2019 mediante 9 entrevistas semiestructuradas a cuidadoras latinoamericanas, que hubieran atendido a personas al final de la vida en Granada (España).

**Resultados:**

Surgieron 2 temas: “Cuidadora migrante al final de la vida” e “Y ahora ¿qué hago?”: El impacto de la pérdida a nivel económico, emocional y laboral.

**Conclusión:**

La atención durante el final de la vida de la persona cuidada genera una sobrecarga adicional a la situación de las mujeres migrantes. La vivencia de esta etapa se relaciona con el vínculo con la persona cuidada y su familia, que puede incidir en la elaboración de un duelo complicado y problemas personales relacionados con la pérdida de empleo y la ausencia de apoyo económico.

## RESUMO

**Objetivo:**

Descrever e compreender a experiência de mulheres migrantes latino-americanas, cuidadoras de idosos em situações de doença avançada e de fim da vida.

**Método:**

Estudo qualitativo baseado na fenomenologia hermenêutica de Gadamer. Os dados foram coletados em 2019 por meio de 9 entrevistas semiestruturadas com mulheres cuidadoras latino-americanas que cuidaram de pessoas no final da vida em Granada (Espanha).

**Resultados:**

Surgiram dois temas: “Cuidador migrante no fim da vida” e “E agora, o que eu faço?”: o impacto da perda nos níveis econômico, emocional e de trabalho.

**Conclusão:**

O cuidado durante o fim da vida da pessoa cuidada gera uma sobrecarga adicional à situação das mulheres migrantes. A experiência dessa fase está relacionada ao vínculo com a pessoa cuidada e sua família, o que pode ter um impacto na elaboração de luto complicado e problemas pessoais relacionados à perda do emprego e à ausência de apoio econômico.

## INTRODUCTION

The aging process in recent years is leading to a significant increase in the number of people over 80 years of age who are unable to care for themselves^(1)^. This reality, together with the increasingly limited availability of family caregivers, has increased the need to hire home care workers to take care of dependent persons^(1)^. In the case of Spain, 20% of the population over 65 years of age is not autonomous and requires assistance for care, and 50% to perform household chores, which is increasing with age^(2)^.

Added to the transformation of the family, it must be added the scarce implementation of formalized social and health services of proximity^(3)^. In addition, the low public budgets allocated to dependency have led to the emergence of a new group of caregivers^(3)^. A total of 356 thousand caregivers of foreign nationality were counted in Spain in 2017, mainly Latin American women^(3)^. This has led to an adjustment of the traditional model of family caregiving, in which caregiving is still feminized and remains within the home^(3)^. All this happens under a new model of commoditized domestic care that is difficult to estimate in actual numbers, since a large percentage are immigrants working in the “gray market” or even without any type of contract^(4)^.

Most foreign caregivers decision to migrate happens for economic reasons^(5)^, coming to work as caregivers in Spain through a relative, friend or association that works with immigrants. The labor reality once in the country is that a large percentage do not have a contract, which leads to the invisibility of women in the migratory processes who, in addition to caring, perform domestic tasks as part of the care and work in-house (interns) or full time in an attempt to cope with the precarious situation of their family economy^(6)^.

The ethno-stratification of work refers to the limitation suffered by immigrants in access to certain types of employment (prioritizing ethnic origins over professional skills). This limitation, together with the feminization of care^(3)^, means that domestic and care work is practically the only employment option for immigrant women. Due to their residency status, they are exposed to situations of special vulnerability and discrimination^(7)^.

For these reasons, end-of-life care provided by people from other countries can cause cultural shocks related to the development of anxiety and other feelings, as well as differences in the way caregiving is understood and, therefore, in how the tasks involved in this process are managed^(8)^ in the caregiver, her family and also in the caregiver herself. This aspect can be of great relevance when care is provided at the end of life, since the experience of death in Latin American countries and in Spain is not the same^(9)^.

On the other hand, caring for patients at the end of life at home involves higher levels of caregiver overload^(10)^, a phenomenon that has been explored in informal caregivers, who experience a complex anticipatory grief that is generally more intense than that experienced after death, due to the loss of the cared-for person during the illness^(11)^.

Latin American women caregivers are an important group in the provision of care who establish a particular relationship with the cared-for person, where the idea of family and the principle of reciprocity prevails, factors that modulate the high personal involvement^(12)^. These are women whose quality of life and access to health services are lower than those of the rest of the population^(7)^. In most cases they lack health training^(7)^ and tend to use complementary therapies based on traditional medicine to a greater extent during the provision of care^(12)^. Their work usually supports their families in their country of origin^(13)^, so the death of the caregiver may imply the end of their work activity.

The experience of migrant caregivers and the families that hire them has been studied in depth^(14)^, but little is known about their experiences in relation to end-of-life care. No studies have been found to date that describe the experiences associated with this process.

The purpose of this study was to describe and understand the experience of Latin American migrant women as caregivers of elderly people in advanced illness and end-of-life situations.

## METHODS

### Study Design

This is a qualitative study, based on Gadamer’s hermeneutic phenomenology. According to Gadamer’s philosophy, the understanding of a phenomenon involves a prior judgment (based on tradition and personal experience, the individual’s history) and the fusion of horizons (the creation of reality in the relationship between individuals), where the result becomes a whole that exceeds the sum of the parts. It is a process that must specify the position of the participants in their context^(15)^.

The study followed the phases as proposed by Fleming et al.^(16)^, translating Gadamer’s philosophy into a method. First, the methodological appropriateness of the research question was agreed upon and then the researchers’ preconceived ideas about the topic were reviewed and identified.

### Population

This study was conducted in the province of Granada (Spain) in 2019. Participants were Latin American immigrant caregivers who had been involved in the care of a person in their end-of- life process. Intentional sampling was performed using the snowball method, a strategy frequently applied in marginalized populations with irregular status such as the one analyzed in this study^(12)^.

### Selection Criteria

Inclusion criteria were: Latin American origin, residing in the province of Granada, having been a caregiver during the End-of-Life Process in exchange for remuneration, and accepting to participate freely and voluntarily. Exclusion criteria included: being minors without the permission of an adult, relatives of the cared-for person, and/or having difficulty remembering data.

The main researcher contacted civil society associations in Granada working with immigrants, by telephone, in person and by e-mail, contacting the people in charge who acted as intermediaries to find potential participants.

### Data Collection

Data were collected through semi-structured interviews, which were conducted between March and June 2019, two of them at the home of the interviewees, while seven were held at the researcher’s home.

Before the start of each interview, a hetero-administered questionnaire of general sociodemographic data and occupational data on their situation as caregivers was completed.

The interviews were based on a common thematic script, exploring the context of the beginning of caregiving and the barriers and difficulties as well as the strengths related to end- of-life care, all with the aim of interpreting meanings from the phenomena described. Thus, the participants were asked about the context in which they began their work as caregivers and the meaning that this situation had in their lives. Likewise, we inquired about aspects related to the bonds established with the caregiver and the impact of their company at the end of life, addressing possible difficulties and support throughout the process.

During the interviews, some participants experienced moments of emotional intensity owing to the contents of the experiences revealed. Those situations were resolved by the interviewer through empathy and active listening. On the other hand, the interviews revealed situations of violation of labor rights, such as illegal residence, highlighting the vital importance of maintaining the anonymity of the participants, which could have negative legal consequences if the precautions mentioned above are not taken. For this reason, the associations through which the interviewed participants were finally recruited are not detailed at any time.

The interviews lasted between 44–120 minutes. After data collection, the interviews were identified by alphanumeric code.

### Data Analysis

The interviews were transcribed for its subsequent analysis using Atlas.ti software (version 7.5.4).

Following the method proposed by Fleming et al.^(16)^, the steps followed for the analysis were as follows: An open reading was carried out, posing the following questions in relation to the text: what influence do sociocultural factors have on the experience of end-of-life care? How do Latin American migrant women migrant caregivers experience the death of the cared- for person? The quotations were initially coded in search of juxtaposition among the participants’ narratives, which occurred with the fusion into units of meaning, grouping representative themes. Coherence emerges with the appreciation of the whole, where each part is important, but to understand them we need this fusion of horizons.

### Rigor

The last stage of the method is meant to establish the reliability and veracity of the process and analysis^(16)^. From the analysis of each sentence, units of meaning, subthemes and themes were identified. The coding of the interviews was carried out by three members of the research team for triangulation. Subsequently, an independent researcher read the transcripts to confirm agreement with the findings of the study, as well as the inclusion of the perspectives of all participants. The researchers interchanged memos during the analysis process to reflect on their pre-understandings and possible impact on the different stages of the research process. To ensure this representation, the data collected and extracted during all stages of the research were confirmed with the participants.

### Ethical Aspects

All participants signed an informed consent form, being previously informed of the purpose of the study and the voluntary nature of their participation. Confidentiality and anonymity were guaranteed throughout the process. The data were treated in accordance with the Spain’s Organic Law 3/2018 on Personal Data Protection and guarantee of digital rights. To preserve the identity of the participants, their names were replaced by the letter “P” (participant) followed by the number according to the order in which they were interviewed.

The study was previously approved with a favorable report by the Research Ethics Committee of the University of Granada, with registration number 775/CEIH/2019.

## RESULTS

**Chart 1.**
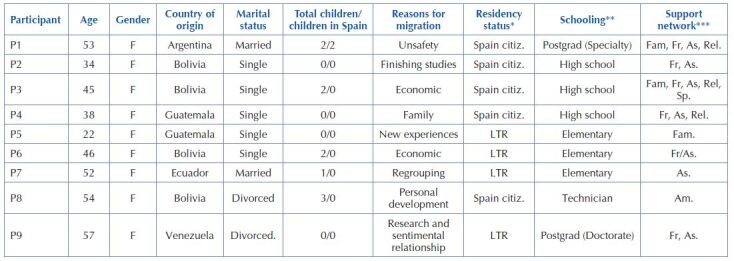
Sociodemographic data – Granada, Spain, 2019.

A total of 12 caregivers were contacted of whom 3 declined to participate due to time constraints and fear of being identified or exposed to the interview. Therefore, the final sample consisted of 9 participants, all women, with a mean age of 44.5 years (Standard deviation: 11.17). A total of 55.5% already had Spanish nationality and 44.44% had long-term residence. 55.5% had children, but only 11.1% lived in Spain. With respect to educational level, approximately half had not had access to higher education, although those who did had completed their education, including postgraduate studies. The sociodemographic data are presented in Chart 1.

*Spain citiz.: Spanish citizenship. LTR: Long-term residency with nationality pending. **Postgrad, Specialty or Doctorate. ***Fam: Family. Fr: Friend. As: Association. Rel: Religious Sp: Sports.

**Chart 2.**
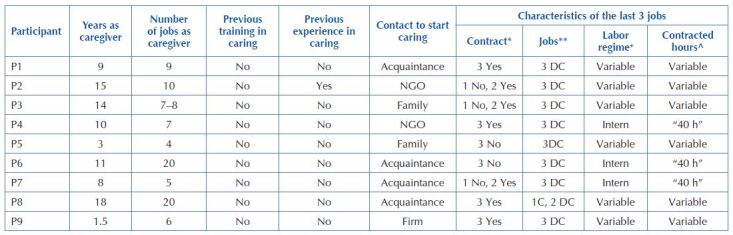
Labor data as caregivers – Granada, Spain, 2019.

Regarding employment data (Chart 2), only one interviewee had previous training after having begun studies as a nursing assistant, however, none had previous experience. At the time of the interview, 11.1% were inactive due to illness, 66.6% were working under contract and 22.2% were working without a contract. 22.2% were working as living-in (interns), 11.1% as semi- interns, 11.1% as full-time interns and the remaining 44.4% on an hourly basis. All had worked as living-ins (interns) at least once.

*Number.: refers to the number of jobs with respect to the modality. No: Without contract. Yes: With contract. **Number.: Number of jobs with respect to the modality. DC: Domestic and care activities. C: Only care. +Variable: Different regime for each of the 3 jobs, varying between: hourly, intern, full-time and others. ^Variable: Different schedule for each of the 3 jobs, ranging from 2 hours to internship. “40h”: Verbalization that there are 40 h per contract, but many more hours actually worked.

With regards to the reasons for migration, each caregiver migrates with a different personal history. Early family deaths and absent figures, divorces, studies and with it, different jobs that affect the way in which they live not only the change of country but also the adaptation to work as caregivers. There is an ideal of what will be achieved by migrating to Spain: regrouping with their family, starting a new life as a couple or a completely new one, studying, researching, learning, saving; however, the expectation is often different from the reality that was achieved in Spain, often leading to regret for having migrated.

**Chart 3.**
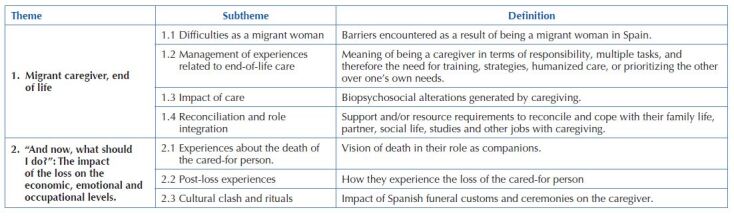
Themes, subthemes and units of meaning – Granada, Spain, 2019.

The results of the analysis of the interviews were organized into units of meaning, subthemes and two main themes (see Chart 3).

*1. Migrant Caregiver, At the End of Life*

Correspond to experiences in the world of care, including the end-of-life period, as a migrant woman.

*1.1 Difficulties as a migrant woman*. Experiences as a caregiver are marked by numerous difficulties. In the first place, the social barrier was detected, on discovering that practically her only job opportunity is caregiving, where she must adapt to a culturally different family, feeling fear, loneliness, uprooting, with pressures of loans, remittances and having to make ends meet in Spain.

*No matter how long I live here, how much I want to adapt to this society, how much I want to get used to their customs, and even though I already have the citizenship, that does not give me any guarantee that I will be seen as an equal. (P2:279)*

*It was said that in Spain there was a lot of work, that they earned very well, but at no time did they say that it was as they say here the Latinos “cleaning butts”, pardon my expression (P3:142).*

This difficulty in accessing certain jobs, together with their migratory situation or status, means that most of them end up working in precarious conditions, without rest, without privacy, without regularity in payments or contracts, without limits on the work to be done or basic implements. The living-in (interns) modality is called “modern slavery” by them, and they are reluctant to work this way, so they change their families.

*Slavery used to exist, but now it is modern because they pay you (P1:115).*

*I only put my feet on the street in my free day, which was Saturday at noon until Sunday night, I worked like that for eight years (P2:20).*

The other barrier detected has to do with the fact of being a woman. This situation puts them in a situation that accentuates the discrimination suffered. In general, they state that migrant men do not do domestic work, are more often chosen to take care of men, have higher pay, gain respect for their free time, recognition of the work done, and strength. Thus, care, like the outside world, perpetuates machismo.

*But he’s a man, he can’t do the dishes. And when it came to payday, he did less than I did, I even had to do his work and my salary was the same and his was more than mine. (P2:263)*

*Men decide about their time, we can’t, others do (P3:140).*

This double discrimination often translates into a risk of suffering mistreatment, whether discriminatory, racist, sexual advances, or the feeling that despite the existence of an affective bond, they are replaceable, which makes some of them feel disposable.

*The husband takes my arm and says “come, sit here with me”, I say “what for?”, and he says “come I want to talk to you” and I say… ok, he’s my boss, or he wasn’t my boss I say ok. We sit down he says “look, since you are here to clean, to satisfy my wife in cleaning the house”, that was his word, “I think you can also satisfy me” and I say, “satisfy you how?”, and he says “well, you are not stupid, you are already grown-up, and you know well what I am talking about”. (P3:162)*

*“Do you want me to denounce you?”, and he says “you are going to lose out… well, you know… here foreigners always lose, even if you have papers, who are they going to believe?, I am the master of the house, I have earned my own reputation in my work, in my family, in my town, who is going to believe you, the way you look, they are going to believe that you are hitting on me” (P3:108).*

*1.2 Management of experiences related to end-of-life care*. In the end-of-life care stage, the caregivers point out experiences such as diagnostic misinformation, the perception of near death, the announcement by the cared-for person that he/she will die, feelings of despair linked to not knowing what to do, and descriptions of their farewells.

*The doctors decided to leave him there to end his days, but of course, no one would tell me. Nobody really told me the story of how it was (P1:163).*

*We got to the hospital, well until she came in. I remember her face; she said goodbye to me (P9:60).*

An important element in managing these experiences has to do with the bonds established with both the cared-for person and his or her family. Many times, the person that receives care chooses and supports the caregiver, generating an affective bond. This awakens a certain duality in the caregiver, since she has changed her focus of affection by having her family absent. On other occasions, there may be difficult coexistence and even aggressiveness. As for the families, the relationship is varied. When the caregiver is not mistreated, the bond with her is one of good treatment and support. On other occasions, there is a lack of empathy towards them, and families are not very involved in the care of their relatives.

*At that moment the lady who was behind me, because, when they started arguing, I put myself between the two of them and she put herself behind me, she put herself in front of the daughter and said “before she leaves”, she was referring to me, right, “you leave”, she said to the daughter. The daughter didn’t know what to say, she turned around, slammed the door and left (P2:305).*

*She had a very acute Alzheimer’s disease, so she started to hit me (P4:103).*

*Now that I have been with this problem, her daughter has been there, she is a nurse, she has been there watching me, calling me, giving me strength and support (P7:33).*

*1.3 Impact of caregiving*. Giving care affects sleep, appetite, mental health, generating pain and changes in dynamics, since they are caregivers, in addition, of their own family at a distance, with guilt for the absence.

*I COULD NOT LIFT HIM! my back was hurting, I felt like I was going to break my back (P3:174).*

*With the Alzheimer’s lady, I had a very bad time, I experienced an anxiety attack (P4:136).*

During the end-of-life stage, the caregiver must also be on call, care becomes more complex, involving constant worry and suffering when seeing the cared-for person suffering. The family often delegates decision-making to them, or, on the contrary, prevents them from participating in them. However, in some cases the interviewees deny overburdening during this period.

*The last time when she saw that, that the mother was also leaving, that was when she started to, like, to, to transfer all the responsibilities on me, “whatever you tell me”, “whatever you decide.” (P1:279)*

*But I had to clean it too, that is, the tube, and if it came out, I had to know how to put it back in. It was complicated. Because she slept with it on and I woke up many times at night, even though she slept all night, I woke up many times because I thought I could move my hand and take it out, things that, and I said “what if I don’t notice, what if I fall asleep, what if something happens, what if it gets infected, what if…” A lot of things that, that you don’t want to happen, but you know that they can happen, so, it was very hard the last time. (P1:45)*

*1.4 Conciliation and integration of roles.* From the experiences and complexity of this phase, there is a rising need to integrate her role as a caregiver and reconcile it with her personal life. They detect needs such as physical support, emotional support and tools, social recognition, courses/training, changes in working conditions, advice on care and support from mental health professionals. In this sense, family, friends, other caregivers and spirituality appear as fundamental support networks. Although institutions provide benefits, sometimes they generate impediments to provide help.

*I did not seek professional help, but I suppose that this would also have to be seen in terms of caregivers, because not only the family suffers, but we do too, because we are much more involved than the family. (P1:297).*

*The one who was with her and saw her die was my husband (1.0) he was my representative, and that is something that I, I will be eternally grateful to my husband, because not just anyone can do that. (P1:190).*

*2. “And now, what should I do?”: The impact of the loss at the economic, emotional and work level*.

This refers to the process of being company to each other during the death of the cared-for person, together with the subsequent mourning that the caregiver elaborates and the way in which she expresses the mourning.

*2.1 Experiences of the caregiver’s death*. In their role as caregivers, these women frequently accompany the death of the cared-for person. They experience it through prayer, omission of the prognosis, or in the context of health care. All this generates their own vision of death, which has an impact on the caregiver’s life at the economic, emotional and work levels, leaving her in a situation of triple unprotection, with no help.

*I didn’t believe that I was going to be the last one to touch his hand, to feel his breath, even though his wife and son were there, but no, he was, he wanted ME to be the one next to him. (P3:52)*

*There is an impressive mix of emotions there. All the experiences you had, the affection you had for that person and the feeling of “and now, what should I do?, what will become of my family, what will become of me without a job” (P1:288).*

*2.2 Experiences after the loss*. This loss is experienced differently mainly according to the support of family members. Many times, they continue to work in the same place, sharing the grief with them. Some of them seem to have unresolved grief, still experiencing it with pain.

*I turned to the lady, she cried a lot, we cried alone, we cried alone (P2:69).*

*They start to say “hey, XXXX ((P4’s name)) get your stuff out soon”, or people don’t have the conscience to tell you “Stay for a week”, I don’t know, “how do you feel”, “stay for a while”, or I don’t know, I don’t know, at least at that moment you don’t are at point zero, so to say. But nevertheless, people don’t give a damn regarding them. “It’s over, that’s it.” (P4:159)*

Despite the experiences reported and the difficulties encountered, caregivers report that migration also has benefits, and caregiving rewards, whereby the overall experience generates learning and reflections that help caregivers develop strategies to move forward. Thus, in order to move forward, caregivers use as strategies to become less involved with the cared-for person, to disconnect, to take care of themselves.

*I think caregiving gives you, well, a point of view, it gives you a great human satisfaction. (P4:153)*

*It is very difficult, isn’t it? To come from far away, to adapt to all this situation, to get attached to people, then to lose them, to stand out from that loss and to make as if your life, mmm, you look for another side, that is, the same thing happens again and you have to recover. In the end, I think you become like a machine, don’t you, that you no longer feel, and it is another job that you have to see it that way, so that it doesn’t affect you, and besides, it is a necessity, you come here from far away, it is because you need to work. (P2:132)*

*2.3 Culture clash and rituals*. Regarding the way in which funeral rites are celebrated in Spain, they state that they are different, with less accompaniment of the deceased, greater speed and simplicity, use of the crematorium, less expression of crying, and a more natural experience of death compared to their countries of origin.

*I did not see them cry, but not because they did not feel, I DO NOT KNOW, I imagine that they do feel too, but for me it was the first time that I saw someone die, and more holding my hand, and on top of that, it could also be that I saw myself reflected with my grandmother, right? And I said “I’m far away”, I don’t know, it was a, an accumulation of sensations, and when we buried her, they took me down again and she told me “We are going to give you another pill”, she told me and I said “no” and she said “yes, yes because you are not well”, and I said “well… give it to me”, they gave it to me but I didn’t take it. (P2:48)*

*So, the crematorium had a huge impact on me (P3:120).*

## DISCUSSION

The main objective of this study was describing and understanding the experience of Latin American migrant women as caregivers of elderly people in advanced illness and at the end of life. It was found that regardless of having Spanish citizenship and whether or not they have a contract, the work they do is not as formal caregivers but as domestic work that includes caring for the sick person, as living-in, full-time or by the hour. This corroborates the ethno-stratification of work, mentioned in the introduction, which characterizes the employment of migrant women in Spain. Ignorance of this reality may explain the mismatch experienced by some women with the expectations created when they decided to migrate and the difficulties in developing the life project associated with this decision^(7)^.

On the other hand, specific stressors related to the migratory experience and the acculturation process^(17)^ have been described, such as the fear of being deported, a social determinant in predicting mental illness, and even used by employers to perpetuate precarious working conditions^(13)^, permanent economic need and family obligations or remittances (sending money to their country of origin), which explain higher levels of depression, anxiety and burnout^(18)^, placing them as migrants in a position of vulnerability at the outset.

The group of interviewed caregivers reported precarious working conditions, such as lack of rest, lack of privacy limits and high levels of social isolation^(19)^. Special attention should be paid to live-in (intern) work, described by the caregivers as “modern slavery”, a term already addressed in the existing literature^(20)^. Migrant live-in caregivers are at greater risk of abuse, exploitation and normalization of the loss of residency status, accepting precarious working conditions with the promise of permanent residency and the possibility of improving their lives along with those of their families^(19)^. The risk of abuse and sexual harassment of foreign caregivers has already been detected in previous studies as sexual advances towards caregivers, in addition to discriminatory and racist treatment^(21)^. Despite these difficulties and all the adversities previously noted, the group of caregivers remains working in care, an aspect addressed in other geographic areas such as New Zealand, where they observed that irregular residence, the pressure to send remittances, and the requirement to keep the job to feel at home, made immigrant caregivers tolerate low wages and long working hours^(22)^, as is the case with part of this group of caregivers.

Regarding their experience with end-of-life care, several issues were found to characterize this experience. First, once the adaptation to living with a culturally different family has been overcome, the bond that caregivers share with the people they care, emerges from this coexistence. This bond has been related to the process of “kinship” that arises at the intersection of formal (immigrant caregiver) and informal (family) care and the relationships that develop between the elderly caregivers and their families and the migrant domestic workers and their families^(23)^. But it can also involve the difficulty of poorly defined emotional boundaries, with demands from the older cared-for person for greater emotional involvement, and with it domination and exploitation^(22)^.

Secondly, gender differences have been noted between male and female migrant caregivers, mainly related to the exclusive dedication of men to caregiving versus the dedication of women to domestic work, and related to the advantages of men for more regulated and professionalized occupations^(24)^, which confirms the need to adopt a gender perspective in the field of caregiving and migration.

Thirdly, the management of humanized care as well as the experiences and emotions linked with end-of-life care have served to show important gaps in the training, information and availability of resources that they have to face. Some have developed motivational strategies as a protective element and others have reported attitudes of self-postponement previously detected in other studies^(25)^. This self-postponement in the context of the demands of fulfilling multiple tasks and the responsibility of caregiving is directly related to the employing family. Two models have been described: the first centered on the migrant caregiver, where family members focus the pressure on her to perform most of the tasks, generating great stress, with little impact on the well-being of the cared-for person; and the second consists of a team care dynamic shared by the caregiver and the family, meaning less stress for the caregiver and better well-being for the cared-for person^(25)^.

Regarding the final stage of life, caregivers report different experiences, from the complete lack of information in one extreme, to the other extreme in which they are warned by the person him/herself, experiencing despair and farewells. This stage is particularly difficult, placing an additional burden on caregivers. Although the existing literature on the end of life refers to family members, they address aspects pointed out by our group, and in that sense, they behave as family caregivers. Some authors describe the severe overload experienced by caregivers of hospitalized patients^(26)^, analogous to that of our caregivers, and other authors highlight the multiple tasks they perform and their need for: support in caregiving, decision making, trauma support, preparation to confront the reality of the dying and grief support^(27)^.

However, for them, the impact of death has emotional consequences, as in the case of family caregivers, but also work and economic consequences. Migrant caregivers experience a bereavement, little described in the literature, whose trajectory is influenced by the support received from the family cared for^(28)^. The caregivers mentioned emotional support, flexibility in leaving home, help in finding a new job and recognition as positive attitudes from the families. On the other hand, they pointed out the culture clash due to the funeral rituals they observed and the difficulty in saying goodbye.

Finally, it is important to refer to the costs of care born by migrant women, that they have reported. The care performed by Latin American women, with long hours of work and responsibilities, far from their loved ones, impacts their lives and those of their families in a negative way^(29)^. There is also a social impact with respect to the transnational social pressures on women to maintain employment, the captivity generated by their contracts, the limits to unionize, the social isolation and lack of privacy they experience^(19)^. As in previous studies, they point out physical needs for more rest^(30)^, emotional and practical help for specific ethnic groups, better access to education through courses that address dementia management, support groups, and support from health professionals^(25,30)^.

However, they have also pointed out a number of opportunities coming from their experience, such as the benefits of their migration, rewards of care and learning.

There are some strategies they have noted for moving forward with caregiving, such as having time for themselves, managing their thoughts, religion, and social support.

### Limitations

The main limitation of this study has to do with the size of the sample. Despite having contacted numerous associations, only 12 people showed interest in participating, with 9 caregivers finally accepting. This issue is understandable from the point of view of the clandestinity under which most caregivers live^(13)^, being the fear of being identified and, therefore, the possibility of having legal consequences, an impediment when accessing the sample. Despite this limitation, interviews were obtained with a wealth of data.

### Advances for the Nursing/Health Field

The figure of the formal caregiver has an inescapable effect on the care economy. This model of care will remain in our society, adjusting to the changes produced mainly after the incorporation of women into the labor market. Knowing the experiences of these women sheds light on the aspects that should be considered from the provision of care from the different devices involved, since the caregiver adopts a role that should be taken into account when attending to training demands, case management and health problems derived from this specific population, as a consequence of the work performed.

## CONCLUSIONS

Care during the end of life of the person that is cared for, generates an additional overload. The reconciliation of tasks, not only of care but often also domestic tasks, with personal life is complex. The experience of this stage is related to the bond with the caregiver and his or her family, which can have an impact on the development of complicated grief and personal problems related to the loss of employment and the absence of economic support. Cultural differences are mainly identified with funeral rituals, generating an impact on the experience of caregivers. These differences can also lead to difficulties in the relationship with the family, mainly due to attitudes of rejection.
